# Outcome and complications in postcardiotomy cardiogenic shock treated with extracorporeal life support – a systematic review and meta-analysis

**DOI:** 10.1186/s12871-025-02898-2

**Published:** 2025-01-17

**Authors:** Romana Maria Kienlein, Ralf Felix Trauzeddel, Nilufar Akbari, Leonello Avalli, Fausto Biancari, Carlotta Sorini Dini, Sabina Guenther, Christian Hagl, Matthias Heringlake, Jochen Kruppa, Timo Mäkikallio, Raphael Martins, Marc Pineton de Chambrun, Ardawan Julian Rastan, Antonio Rubino, Floris van den Brink, Michael Nordine, Sascha Treskatsch

**Affiliations:** 1 Charité - Universitätsmedizin Berlin, corporate member of Freie Universität Berlin and Humboldt-Universität zu Berlin, Department of Anesthesiology and Intensive Care Medicine, Campus Benjamin Franklin, Hindenburgdamm 30, Berlin, 12203 Germany; 2 Charité – Universitätsmedizin Berlin, corporate member of Freie Universität Berlin and Humboldt-Universität zu Berlin, Institute of Biometry and Clinical Epidemiology, Charitéplatz 1, Berlin, 10117 Germany; 3 Department of Cardiovascular Surgery, Centro Cardiologico Monzino, Milan, Italy; 4 Department of Cardiovascular Surgery, Centro Cardiologico Monzino IRCCS, Milan, Italy; 5 Intensive Cardiac Care Unit, Azienda Ospedaliero-Universitaria Careggi, Florence, Italy; 6 Intensive Cardiac Care Unit, Azienda Ospedaliera-Universitaria Senese, Siena, Italy; 7 Department of Cardiac Surgery, University Hospital Munich, Ludwig-Maximilian-University, Munich, Germany; 8 DZHK (German Centre for Cardiovascular Research), Partner Site Munich Heart Alliance, Munich, Germany; 9 Department of Anesthesiology and Intensive Care Medicine, Heart- and Diabetes Center Mecklenburg - Western Pomerania, Karlsburg Hospital, Karlsburg, Germany; 10 Hochschule Osnabrück, University of Applied Sciences, Osnabrück, Germany; 11 Department of Medicine, South Karelia Central Hospital, University of Helsinki, Lappeenranta, Finland; 12 1CHU Rennes, Service de Cardiologie Et Maladies Vasculaires, Rennes, France; 13 Sorbonne Université, Assistance Publique-Hôpitaux de Paris (AP-HP), Hôpital La Pitié-Salpêtrière, Service de Médecine Intensive Réanimation, Paris, France; 14 Sorbonne Université, Inserm, UMRS_1166-ICAN, Institut de Cardiométabolisme Et Nutrition (ICAN), Paris, France; 15 Department of Cardiac and Thoracic Vascular Surgery, Marburg University Hospital, Marburg, Germany; 16 Department of Anaesthesia and Intensive Care, Royal Papworth Hospital, Cambridge, UK; 17 Department of Intensive Care, Leids Universitair Medisch Centrum, Albinusdreef 2, Leiden, 2333ZA The Netherlands; 18 Department of Anesthesiology, Intensive Care Medicine, and Pain Therapy, University Hospital Frankfurt, Goethe University, Frankfurt, Germany

**Keywords:** Postcardiotomy cardiogenic shock, Outcome, Extracorporeal life support, Veno-arterial extracorporeal membrane oxygenation, Meta-analysis

## Abstract

**Background:**

Postcardiotomy cardiogenic shock (PCCS) in cardiac surgery is associated with a high rate of morbidity and mortality. Beside other therapeutic measures (e.g. intraaortic balloon pump (IABP)), extracorporeal life support is being increasingly used in this particular form of shock. Objectives of this meta-analysis were to determine mortality and complications of extracorporeal life support treatment (ECLS) in cardiac surgery patients, and if outcomes were influenced by a preexisting cardiovascular risk profile.

**Methods:**

MEDLINE and EMBASE were searched for studies in English, published between January 1^st^ 2000 and January 16^th^ 2023, reporting mortality and morbidity in patients aged ≥ 18 treated with ECLS for PCCS. Supplementary data were requested from the respective corresponding authors. Outcomes were weaning from extracorporeal life support, hospital survival and complications.

**Results:**

Two thousand, seven hundred seventy-four papers were screened, of which 132 full text articles were assessed for suitability. 70 remaining studies were included for further evaluation and data analysis. Five studies could be included in the final analysis since the corresponding authors provided additional necessary information. Successful weaning from extracorporeal life support was accomplished in 52.8% (30.8%—57.4%) and 31.1% were discharged alive (mortality of 25.0 – 56.2% after weaning). 95.1% of all treated patients suffered from at least one complication. Diabetes mellitus and obesity seem to be independent risk factors for poor outcome.

**Conclusions:**

Extracorporeal life support for PCCS is associated with a substantial mortality and complication rate. Diabetes mellitus and obesity seem to be independent risk factors. Therefore, until future work has elucidated which patients benefit at all, the risks of ECLS-treatment must be critically weighed up against a possible benefit.

## Background

Postcardiotomy cardiogenic shock (PCCS) is defined as a refractory cardiogenic shock after cardiac surgery—often associated with the inability to wean the patient from cardiopulmonary bypass (CPB)—as a result of insufficient cardiac function resulting in inadequate tissue as well as organ perfusion and oxygenation. PCCS occurs in approximately 2–6% and is associated with a high rate of morbidity and mortality [1–6]. In this context, the use of mechanical circulatory support (MCS) systems, e.g. extracorporeal life support (ECLS) or, often also named, veno-arterial extracorporeal membrane oxygenation (vaECMO), offers a potential treatment option to save patients´ lives and is one of the main indications nowadays for an ECLS therapy [6, 7]. In contrast to other forms of MCS, it offers additional respiratory support, and due to the further development of the devices it is widely available and easy to implement [8]. Absolute ECLS contraindications are irreversible neurological conditions (e.g., brain death, severe encephalopathy), terminal malignancy and the unavailability of consecutive left ventricular assist device (LVAD) implantation or heart transplantation. Some other reasons are considered relative and are mostly based on local standards [9].

In line with current guidelines recommending a more liberal ECLS therapy [9, 10], there has been a three-fold increase in ECLS treatment over the last decade. However, increased ECLS use in order to save patients´ lives was though associated with only little change of morbidity and mortality rates [11–18]. Evidence from randomized trials [19–22] and contradicting effects on outcomes reported in recent meta-analyses [23, 24] question ECLS treatment in the setting of myocardial infarction-associated cardiogenic shock. In this context, a call for improvement in patient selection has grown [6]. Whether baseline characteristics such as age, gender, or obesity beside traditional risk factors (e.g. surgical indication, type of surgery etc.) have an influence on ECLS outcome is also poorly examined. Therefore, primary and secondary objectives of this meta-analysis were to determine mortality and complications of ECLS–treatment in patients with PCCS, and if outcomes were influenced by a preexisting cardiovascular risk profile, respectively.

## Methods

### Literature search strategy and selection criteria

This meta-analysis was approved by the local ethics committee at Charité – Universitätsmedizin Berlin (EA4/239/19) as well as the local data protection authority. Consent to participate was not applicable. On January 16th, 2023, the databases MEDLINE and EMBASE were searched for original published studies published between January 1st 2000 and January 16th 2023. Inclusion criteria were all prospective and retrospective interventional/observational as well as randomized controlled studies (RCT) on adult patients (aged ≥ 18 years) being treated with ECLS for PCCS published in English language. To achieve optimal search accuracy identifying all possible publications the terms “ECLS” or “ECMO” or “extracorporeal life support” or “extracorporeal membrane oxygenation” AND “shock” or “arrest” or “cardiogenic shock” or “cardiac arrest” or “heart” were combined as MESH terms as well as key words. The search string for the databases MEDLINE and EMBASE were as follows:

- MEDLINE: ECLS[All Fields] OR ("extracorporeal membrane oxygenation"[MeSH Terms] OR ("extracorporeal"[All Fields] AND "membrane"[All Fields] AND "oxygenation"[All Fields]) OR "extracorporeal membrane oxygenation"[All Fields] OR "ecmo"[All Fields]) AND (("heart"[MeSH Terms] OR "heart"[All Fields]) OR (("heart"[MeSH Terms] OR "heart"[All Fields] OR "cardiac"[All Fields]) AND ("shock"[MeSH Terms] OR "shock"[All Fields]) OR "arrest"[All Fields])) AND ("humans"[MeSH Terms] AND "adult"[MeSH Terms]).
- EMBASE: ((ECMO or ECLS) and (Cardiac or shock or arrest or heart)).mp. [mp = title, abstract, heading word, drug trade name, original title, device manufacturer, drug manufacturer, device trade name, keyword, floating subheading word, candidate term word].

To ensure that all eligible studies were included, the process of literature search, screening of abstracts and the detailed content of the published papers were conducted as a dual control principle with two reviewers (RFT and RMK). Afterwards the references of all publications were screened to detect all further eligible studies. Furthermore, websites of DRKS (German Clinical trials register) and ClinicalTrials.gov were checked for ongoing studies in this field while drafting this paper.

### Data extraction and quality assessment

For all included studies, supplementary data were requested from the respective corresponding authors by providing a datasheet with definitions for all demanded information to be able to find correlations between patient risk factors and outcomes of ECLS. The non-provision of requested anonymized core data was an additional exclusion criterion. The following data regarding each patient were requested from the authors: patient specific data, pre-ECLS medical conditions, causes and indication of ECMO implantation and therapy, choice of access, implantation site of ECLS, possible additional assist devices, patient parameters before implantation, duration of ECLS therapy, ECLS complications, hospital length of stay as well as survival rates and possible causes of death.

The quality of all included studies was assessed by a newly generated risk of bias tool derived from ROBINS-I tool for non-randomized studies, as there does not exist any general standard quality evaluation tool for non-randomized, non-controlled studies [25]. The following aspects were checked for each study:

- Criteria: Clearly defined in-/exclusion criteria, definition of shock
- Selection: Did all patients meet all inclusion criteria? Were all of them chosen based on the same selection criteria?
- Missing data: < 1% (green), 1—2% (yellow), > 2% (red)
- Outcome: Objective parameters of outcome measurement (e.g. definition of „weaning success”)
- Reporting: Was there a discrepancy between the definitions in the methods part and the results of the outcomes? Is there an indication of selective reporting?
- Conflict of interest (COI): None (green), declaration without COI (yellow), not stated or COI reported (red)
- Analysis data: All present (green), ≥ 7 (yellow), < 7 (red)

### Outcome variables

To establish comparability between the acquired data of the different studies and simplify statistical analysis steps, some parameters were combined within a variable (e.g., combination of liver failure and abdominal organ vessel ischemia to gastrointestinal complications). The endpoints were graduated into primary outcomes (weaning from ECLS and hospital survival) and secondary outcomes (complications).

The detailed definitions of the evaluated endpoints are as follows: weaning from ECLS (defined as successful separation from ECLS without reinsertion or death in the following 24 h), hospital survival, bleeding complications, neurological complications, overall complications. The explanatory variables were smoking, age, gender, arterial hypertension, diabetes mellitus and obesity.

### Statistical analysis

Data and figures were analyzed and generated with R (R Core Team, Vienna Austria, Version 3.6.1.). Demographic and baseline characteristics were analyzed descriptively. For continuous variables, descriptive statistics included median and interquartile range (IQR). For categorical variables, statistics included absolute and relative frequencies. Missing data were investigated in each data set, no imputation was done.

Odds ratios (OR) were calculated for the primary endpoints to compare patients with and without certain risk factors. Ratios were expressed with 95% confidence intervals (CI). The aim was to build two descriptive models to investigate the association of the risk factors with the outcome variables. A one-stage approach was performed, in which all individual patient data (IPD) were summarized into one single dataset that formed the basis for logistic regression analysis. To control for clustering, the study IDs were included as random intercepts. The variables considered are the following known risk factors: diabetes (no/yes), smoking (no/yes), arterial hypertension (no/yes), obesity (no/yes), and age (years). The models were additionally adjusted for sex (female/male). Age was flexibly modelled with restricted cubic splines with two degrees of freedom. Interactions between age and the categorical risk factors were investigated. Multicollinearity was not examined since only one continuous variable was included in the model. Heterogeneity was investigated with the intraclass correlation coefficient (ICC). The two-stage approach was not considered due to problems occurring from low events-per-variable (EPV) ratios resulting from low sample sizes in some studies.

## Results

### Literature research and request of supplementary data

**Fig. 1:**
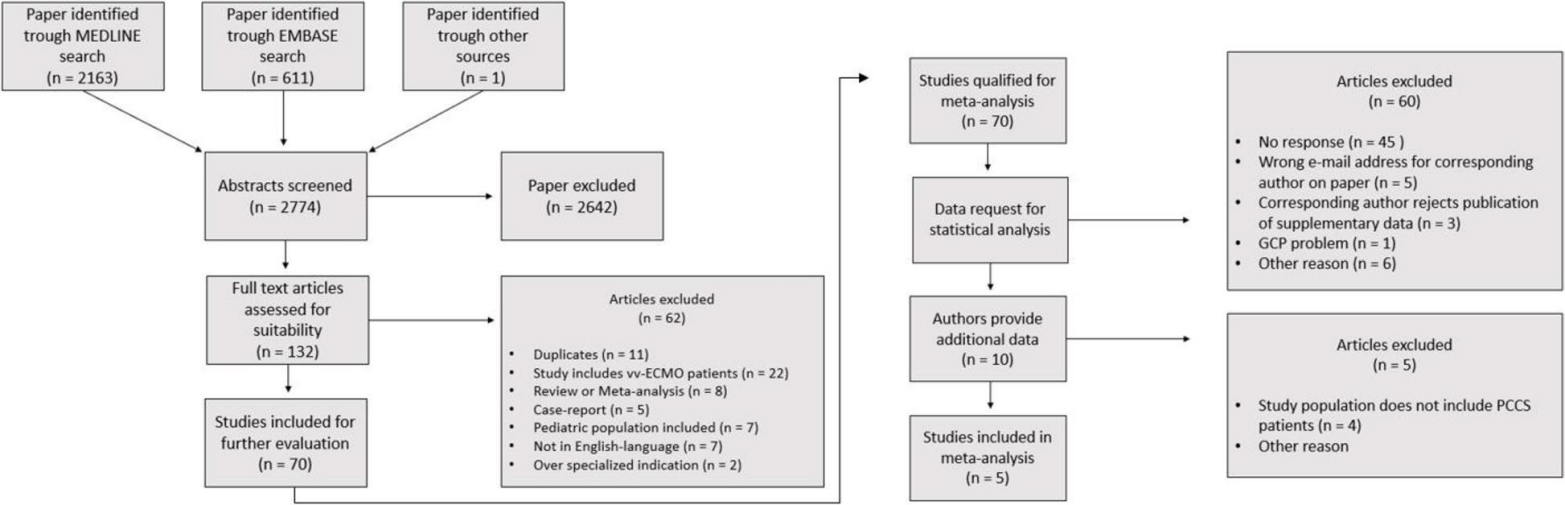
Flowchart of the literature research process and supplementary data request

In total, 2774 papers were screened, of which 132 full text articles were assessed for suitability. All corresponding authors of qualified papers were contacted via e-Mail with the request of delivering patient specific supplementary data. The hand-in deadline for the authors was February 2023 for the update of the data. Five papers were included in the final analysis [26–30] (Fig. 1).

### Quality of studies included in the meta-analysis

The results of the quality assessment are shown in Table 1. Though reporting survival rates to be included in the descriptive analysis, the study by Biancari et al. [27] could not be included in the regression analysis due to the fact that two risk factors (arterial hypertension and smoking) were not reported at all. From the remaining 642 patients, 632 (98.4%) were included in the regression analysis. Only ten patients could not be considered due to missing data. None of the other authors of included studies stated a conflict of interest. Overall, the quality of the included studies was satisfactory.

**Table 1:**
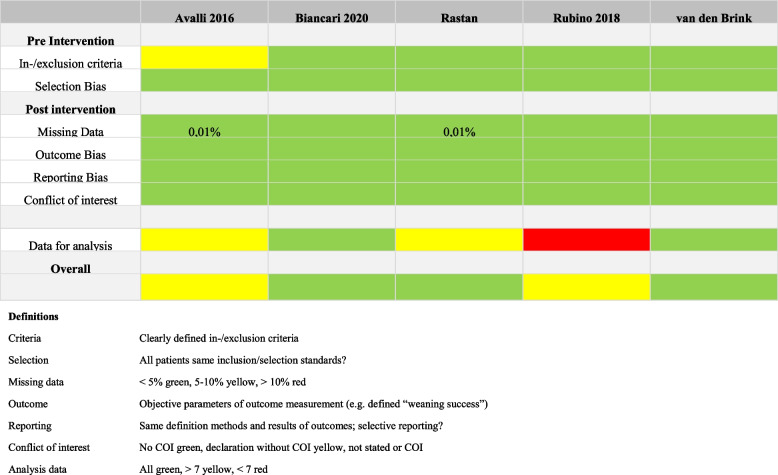
Risk of bias assessment

### Demographic data and descriptive statistics of outcome

The demographic data of the studies and the included patients are shown in Table 2. The descriptive statistics of the analysed outcome parameters are depicted in Table 3. In total, 1307 patients (68.4% male) were included in this analysis. The median age of the patients spreads from 59.0 [31] to 66.7 years [26, 27].

**Table 2:** Demographic data of the included studies

	**Overall**	**Avalli**	**Biancari**	**Rastan**	**Rubino**	**van den Brink**
Year		2016	2020	2010	2018	2019
Study period		2011 – 2014	2010—2018	1996 – 2008	2008 – 2016	2012 – 2016
n	1,307	11	665	517	101	13
Age [years] (median (IQR))	64.6 [55.2, 71.0]	66.7 [63.9, 73.3]	65.4 [56.1, 71.4]	64.1 [55.1, 70.6]	59.0 [48.0, 69.00]	62.0 [55.0, 71.0]
Male (%)	894 (68.4)	4 (36.4)	448 (67.4)	370 (71.6)	64 (63.4)	8 (61.5)
BMI (median (IQR))	26.7 [24.0, 29.7]	NR	26.7 [23.9, 29.9]	26.5 [24.0, 29.3]	27.0 [24.5, 29.7]	26.8 [24.8, 32.8]
Obesity (%)	304 (23.4)	2 (18.2)	165 (24.8)	109 (21.2)	23 (23.7)	5 (38.5)
aHt (%)	389 (61.1)	7 (63.6)	NR	340 (66.4)	34 (33.7)	8 (61.5)
Hyperlipidemia (%)	268 (42.8)	NR	NR	234 (45.7)	24 (23.8)	10 (76.9)
DM (%)	339 (26.0)	2 (18.2)	157 (23.6)	159 (31.0)	18 (17.8)	3 (23.1)
Smoking (%)	167 (26.2)	4 (36.4)	NR	135 (26.4)	28 (27.7)	0 (0.0)
Cardiac condition (%)	520 (65.8)	3 (27.3)	415 (62.4)	NR	93 (92.1)	9 (69.2)

*BMI* Body mass index, *aHt* Arterial hypertension, *DM* Diabetes mellitus, *IQR* Interquartile range, *NR* Not reported

**Table 3:** Overview of the outcome parameters of the included studies

	**Overall**	**Avalli**	**Biancari**	**Rastan**	**Rubino**	**van den Brink**
Duration of ECLS [days] (Median (IQR))	4.0 (2.0; 7.0)	NR	5.3 (2,5; 9.7)	2.0 (1.0;5.0)	6.0 (3.0; 9.0)	5.0 (3.0; 7.0)
Weaning (%)	690 (52.8)	4 (36.4)	332 (49.9)	292 (56.5)	58 (57.4)	4 (30.8)
Hospital survival (%)	406 (31.1)	2 (18.2)	240 (36.1)	128 (24.8)	33 (32.7)	3 (23.1)
Vascular complications (%)	60 (7.6)	5 (45.5)	53 (8.0)	NR	2 (2.0)	0 (0.0)
Ischemic complications (%)	53 (4.1)	3 (27.3)	3 (0.5)	30 (5.8)	16 (15.8)	1 (7.7)
Local infection (%)	78 (10.0)	NR	63 (9.5)	NR	14 (13.9)	1 (7.7)
Bleeding Complications (%)	929 (71.1)	7 (63.6)	458 (68.9)	417 (80.7)	43 (42.6)	4 (30.8)
ECLS circuit complications (%)	146 (11.3)	NR	54 (8.1)	75 (14.5)	16 (15.8)	1 (7.7)
Renal failure (%)	915 (71.0)	2 (18.2)	528 (81.6)	306 (59.2)	66 (65.3)	13 (100.0)
Respiratory complications (%)	522 (40.3)	NR	252 (37.9)	231 (44.7)	35 (34.7)	4 (30.8)
Abdominal complications (%)	563 (43.5)	NR	315 (47.5)	212 (41.0)	36 (35.6)	0 (0.0)
Neurological complications (%)	276 (21.1)	1 (9.1)	124 (18.7)	120 (23.2)	27 (26.7)	4 (30.8)
Overall Complications (%)	1239 (95.1)	9 (81.8)	641 (97.0)	489 (94.6)	87 (86.1)	13 (100.0)

*IQR* Interquartile range, *NR* Not reported

**Fig. 2:**
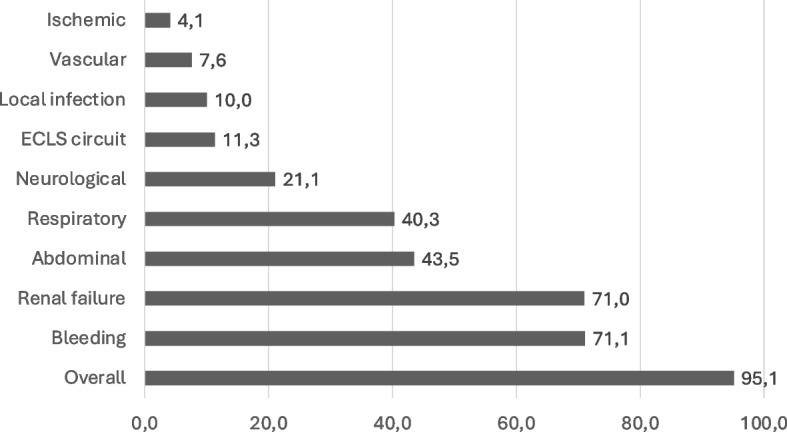
Complications averaged over all included studies

Averaged over all studies, 1239 patients (95.1%) suffered from at least one type of complication with bleeding complications (71.1%) being the most reported one, followed by renal failure (71.0%) and abdominal complications (43.5%) (Fig. 2).

### Weaning from ECLS

**Fig. 3:**
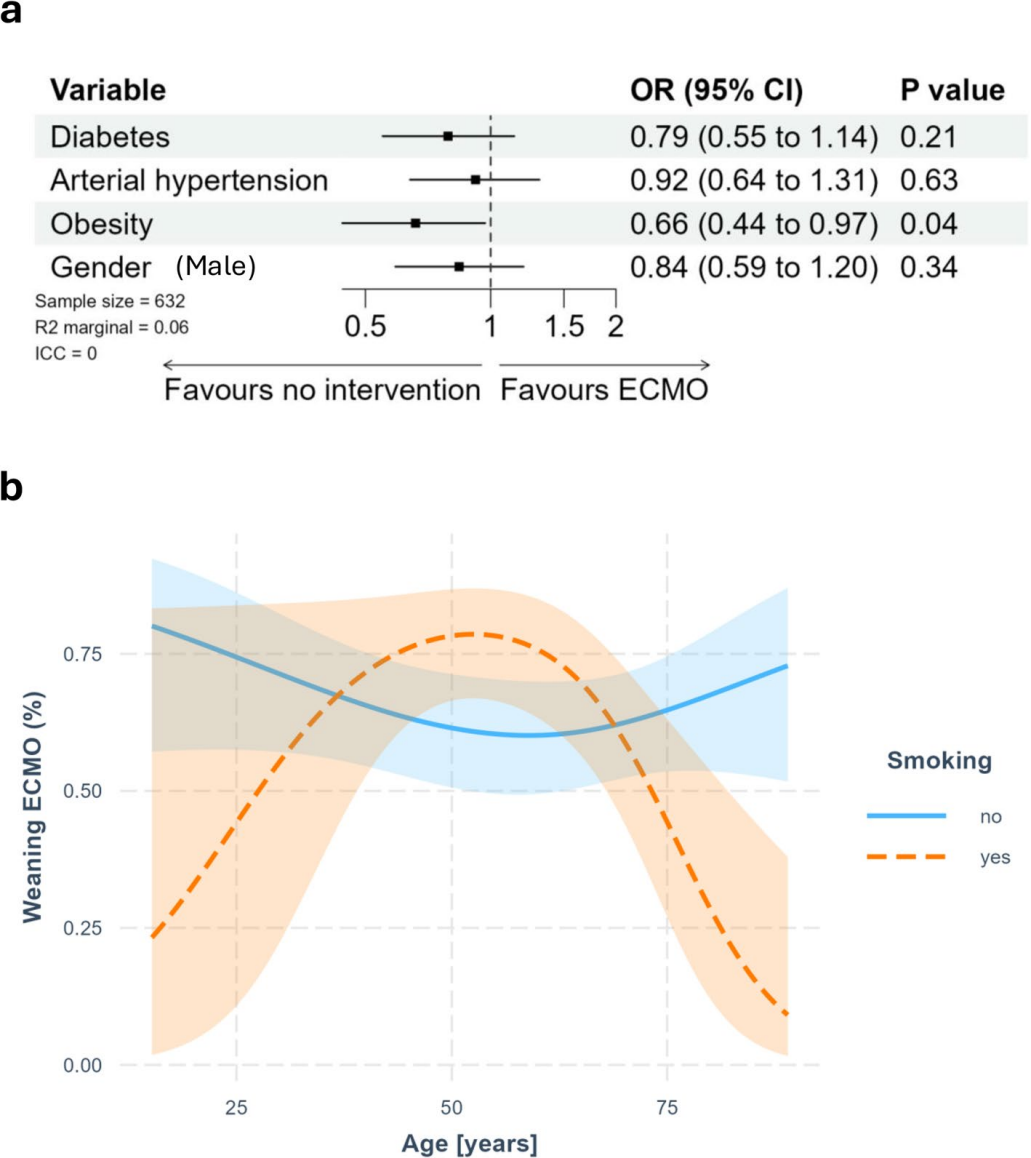
Weaning from ECMO. **a** displays the Forest plot showing odds ratios regarding weaning from ECMO related to risk factors. **b** displays the chart showing the interaction between age, smoking status and weaning from ECLS

Successful weaning from ECLS was accomplished in 52.8% (30.8%—57.4%) of all patients. Patients with hypertensive disease as well as with diabetes had no significant difference in weaning compared to normotensive (OR 0.92; 95% CI 0.64—1.31) or non-diabetics (OR 0.79; 95% CI 0.55—1.14). However, obese patients (BMI > 30 kg/m^2^) had lower odds regarding successful weaning from ECLS compared to non-obese patients (OR 0.66; 95% CI 0.44 – 0.97). Gender specific analysis showed a non-significant tendency towards lower weaning success in men (Fig. 3a).

Regarding ECLS weaning there was an interaction between smoking status and the age of the patients. Smokers showed a tendency to unsuccessful weaning especially when they were younger (< 37,5 years) or older (> 70 years). In contrast, middle aged patients seem to have a higher weaning success rate, in case they were active smokers before ECLS treatment (Fig. 3b). The estimated marginal *R*^2^ value is approximately 0.06, the conditional *R*^2^ could not be determined due to the reduced number of studies.

### Hospital survival

**Fig. 4:**
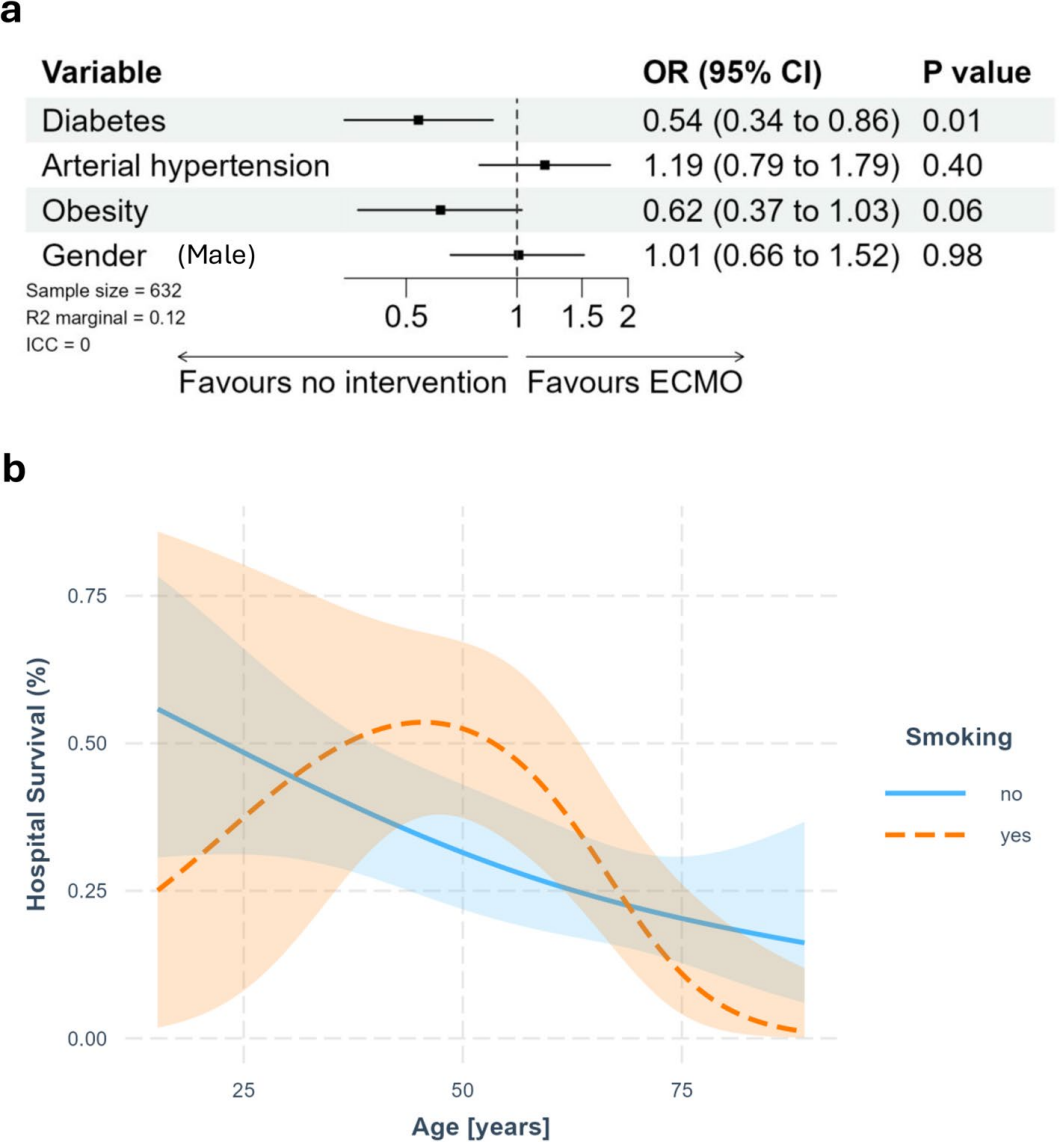
Hospital survival after ECMO therapy. **a** displays the Forest plot showing odds ratios regarding hospital survival related to risk factors. **b** displays the chard showing the interaction between age, smoking status and hospital survival

Hospital survival was 31.1% (18.2—57.4%), resulting in an in-hospital-mortality rate of 25.0—56.2% after successful ECLS weaning. Diabetic patients showed lower OR for hospital survival compared to non-diabetic patients (OR 0.54, 95% CI 0.34—0.86). In addition, obese patients tended towards a lower hospital survival (OR 0.62, 95% CI 0.37—1.03). Hypertensive patients as well as gender did not show any differences in overall hospital survival rate (Fig. 4a). However, there was an interaction between smoking status and age. In non-smokers hospital survival decreased with increasing age, while middle aged smokers were more likely to survive the hospital-stay compared to younger and elderly smokers. (Fig. 4b). The estimated marginal *R*^2^ value is approximately 0.12, the conditional *R*^2^ could not be determined due to the reduced number of studies.

## Discussion

The present study aimed to explore outcomes (weaning, hospital survival) and complication rates of patients treated with ECLS for PCCS. Although heterogeneity was present in the analyzed cohort, high mortality and complication rates were found which were in line with general published ECLS outcome data [11–18]. We found various tendencies regarding influences of cardiovascular risk on ECLS outcome with diabetes mellitus and obesity seeming to be independent risk factors.

ECLS is increasingly used as a rescue therapy for severe low cardiac output syndrome after cardiac surgery as a bridge-to-recovery or bridge-to-decision [8]. Interestingly, mortality rates have not improved or even got worse [8, 32, 33]. Some authors argue that the reason for this observation may be the wider spread of ECLS use in high-risk patients [8]. Interestingly, recent recommendations to initiate ECLS have also become more liberal, despite the lack RCT comparing ECLS with other modalities of mechanical circulatory support in PCCS. Also, there are only a handful of retrospective observational studies demonstrating a benefit of this therapeutic approach. For example, the S3 German practice guideline states that ECLS “may be considered” in PCCS, despite the literature predominantly reports excess mortality in patients treated with ECLS [34].

It remains, however, unclear whether poor outcomes associated with ECLS treatment of PCCS are a result of disease severity vs. treatment technology. While PCCS has a high mortality risk per se, it is still of debate, with the notable exception of patients with life-threatening hypoxia [35], if the use of ECLS is not only a “possible”, but a “useful” treatment modality in comparison with sophisticated medical therapies and/or other types of MCS. In this regard, it is of note that a recent large study from a German register, including more than 380.000 patients with cardiogenic shock, showed the lowest mortality rate amongst patients treated with an intraaortic balloon pump (IABP), while patients treated with ECLS had the highest mortality [36]. Furthermore, it needs to be taken into consideration that—at least in certain health systems (i.e., in Germany)—the high reimbursement of ECLS may trigger the preferential use of this technology despite its unproven effects on outcome. In addition, patients undergoing ECLS treatment show a high-risk of increased left ventricular filling pressures with the need of left ventricular unloading, which frequently is achieved by IABP or microaxial LVAD systems [37, 38]. If a combination of different MCS devices might offer a benefit is not perfectly clear. A recent meta-analysis including 2251 patients did not find a benefit of combining ECLS with IABP in patients with PCCS, neither regarding weaning from ECLS nor hospital survival [16]. In contrast, in the setting of cardiogenic shock a combination of ECLS with IABP revealed a significant mortality reduction [39].

In the last decade, in addition to an increase in the use of ECLS, there has also been a dramatic increase in the use of other mechanical circulatory support devices such as microaxial flow pumps (e.g. Impella) in patients being treated for PCCS. Studies have shown that this method of temporary mechanical support is safe and bears a lower risk of complications while achieving better outcomes regarding weaning from the assist device and hospital survival [40]. Nevertheless, the use of an Impella device alone is sometimes insufficient. As ECLS lacks left ventricular afterload reduction, which is why a combination of Impella devices and ECLS is now often employed. However, the available data on this primarily stem from conservative treatment contexts (e.g. for acute myocardial infarction or pulmonary embolism), making it challenging to generalize these findings for cardiac surgery patients. Furthermore, Impella is typically approached with caution in some patients who have recently received a new aortic valve, aortic root, or aortic arch replacement, due to high risk for complications (e.g. mechanical damage due to the catheter, risk for bleeding or thrombosis, impairment of valve function) [41].

To further investigate the role of ECLS devices compared to other mechanical support devices in terms of invasiveness, outcomes, complication rates, possible respiratory support, costs and long term quality of life aspects, more comparative analyses are needed to optimize treatment strategies for patients treated for PCCS.

ECLS treatment may thus only be considered under strict precautions [42]. This general recommendation is based on a systematic review and collaborative meta-analysis of randomized trials published in 2017 by Thiele et al. comparing the outcome of active percutaneous MCS such as ECLS and another comparator (IABP) for patients in cardiogenic shock (CS) due to myocardial infarction. In that study 46.8% of MCS patients suffered bleeding complications and exhibited a limb ischemia rate of 16.9%. Based on these findings, the authors stated that MCS should be restricted to a select patient group and should be carefully considered due to excessive complication rates [19, 43]. Recently, a randomized controlled multicentre trial investigating the role of early routine ECLS treatment compared to usual medical therapy in patients with acute myocardial infarction complicated by cardiogenic shock and early myocardial revascularization could also not show a survival benefit for the application of ECLS or differences in time to hemodynamic stabilization between both groups [22]. It was postulated that one of the reasons for the absence of a benefit was the significantly higher rate of complications in the ECLS group, namely bleeding and peripheral vascular complications [22] which is also supported by our analysis. The study by Thiele et al. is also in accordance with other randomized trials on the role of ECLS in cardiogenic shock where a lack of improvement in morbidity and mortality was demonstrated [21, 44, 45]. Moreover, a recent meta-analysis investigating the early routine use of ECLS in infarct-related CS compared to optimal medical therapy alone consisting of four randomised clinical trials could not show a reduction in 30-day mortality rates with the use of ECLS, but higher rates of complications for major bleeding and peripheral ischaemic vascular complications [46]. Though PCCS may not be interchangeable with myocardial infarction related CS, both pathophysiological pathways include acute loss of myocardial/contractile function despite causative therapy leading to inadequate tissue oxygenation and systemic inflammation promoting the disease.

Various authors have now advocated for an evaluation of risk factors that may indicate poor outcomes of ECLS in PCCS [6, 47]. In a retrospective multicenter cohort study factors associated with reduced hospital survival were age and preoperative cardiac arrest [48]. In our analysis we found diabetes mellitus and obesity as independent risk factors. However, we were also able to demonstrate an interaction of smoking status and age to have an influence both on weaning from ECLS and hospital survival. Nevertheless, it cannot be ruled out, that these findings were findings by chance. Yet, this finding may trigger further research and requires additional studies.

For both endpoints (weaning and hospital survival) the wide confidence interval for younger patients with an active smoking status is related to the small sample size of this patient collective, leading to a difficult estimation. However, this finding is in line with an analysis including > 15.000 patients in the registry of the Extracorporeal Life Support Organization showing an association between age (as early as 40 years of age) and the occurrence of death and complications [49]. Other factors, e.g. arterial hypertension and gender showed only a tendency regarding ECLS outcome. Recent meta-analyses and systematic reviews on ECLS in patients with cardiogenic shock showed similar results in their findings of weaning and survival outcomes [14, 50, 51]. Our study was able to support this data. ECLS therapy even in specialized centers still incorporates a high risk profile.

There was substantial heterogeneity in the outcomes between the different studies included in our analysis. We tried to assess this heterogeneity in the form of intra-class correlation coefficients. Due to the low number of studies included in the regression analysis, the variance for the random effect was estimated at zero. Furthermore, we report the R2 value as marginal. This R2 value indicates how much of the variance of the dependent variable (outcome) can be explained by the independent variables. It lies between 0 and 1. A higher value indicates a better model. The overall relatively low R2 values in our analyses might be an indication of unexplained remaining variances. One possibility might be that other factors also play an important role in determining ECLS outcome.

### Limitations

The present analysis has important limitations:

1. A main limitation of this meta-analysis is the small number of included studies, since only five out of 70 eligible studies at that time could be included due to poor resonance for supplementary data request needed for analysis. This implies selection bias in the study group. If the summary of the best available evidence based on representative and high-quality studies is not given, several risks as biased estimation of treatment effects, lack of generalizability of the results or outcome reporting bias can arise. Selection bias is a serious problem especially in meta-analyses because it can significantly affect the generalizability and reliability of the results and therefore the conclusions drawn from those.
2. We only used patient data for our analysis instead of using all reported data of the studies. It was our aim to examine whether outcomes of patients with PCCS treated with ECLS were influenced by a preexisting cardiovascular risk profile. However, by relying on the individual patient data, we compromised on the possible number of patients included in our analysis and possibly risked selection bias.
3. An in-depth analysis of the included studies revealed an overall high quality. Nevertheless, there was inhomogeneous reporting of outcome parameters between the studies, which compromised the ability to thoroughly analyse and compare this data. Also, the sample size of patients included in the five studies ranged from 11 to 665. Thus, even if the studies were weighted initially before conducting the forest plots and further statistical analyses, there is still a risk of skewed results.
4. Despite that all included studies were performed in experienced centres, there was a large variability in mortality and complications rates. This variability may be due to heterogeneity between the different centers regarding patient selection, management of ECLS treatment (i.e., by using a modality to unload the left ventricle), or concomitant treatments. However, this could not be derived from the available data and may be regarded as an important limitation. In addition, selective reporting bias should be considered as the clinical outcomes have not been consistently reported among the different studies. Furthermore, it was not possible to analyse further factors (e.g. kind of cardiac surgery and urgency, causes of death or weaning failure, duration of ECLS support) that might influence the outcome of ECLS treatment, as not all studies were able to provide this data.
5. A further limitation may present the timeline of the performed search as especially in more recent times new devices and/or combinations (e.g. Impella and ECLS) arised.

## Conclusions

Extracorporeal life support for PCCS is associated with a substantial mortality and complication rate with diabetes mellitus and obesity seeming to be independent risk factors. Therefore, until future work has elucidated which patients benefit at all, the risk of ECLS-treatment must be critically weighed up against a possible benefit.

## Data Availability

The datasets presented in this article are not readily available because of German Data Protection laws. Requests to access the datasets should be directed to the corresponding author.

## Abbreviations

BMI: Body mass index
CI: Confidence interval
COI: Conflict of interest
CPB: Cardiopulmonary bypass
CS: Cardiogenic shock
DRKS: German clinical trials register
ECLS: Extracorporeal life support
EPV: Events-per-variable
IABP: Intraaortic balloon pump
ICC: Intraclass correlation coefficient
IPD: Individual patient data
IQR: Interquartile range
LVAD: Left ventricular assist device
MCS: Mechanical circulatory support
OR: Odds ratios
PCCS: Postcardiotomy cardiogenic shock
RCT: Randomized controlled studies
va ECMO: Veno-arterial extracorporeal membrane oxygenation
